# Comparison of ExPress Implantation and Partial Deep Sclerectomy Combined with ExPress Implantation and Simultaneous Phacoemulsification

**DOI:** 10.1155/2019/7424376

**Published:** 2019-11-28

**Authors:** Marietta Frączkiewicz-Skok, Joanna Konopińska, Zofia Mariak, Marek Rękas

**Affiliations:** ^1^Department of Ophthalmology, Military Institute of Medicine, Szaserów 128 STR, 04-141 Warsaw, Poland; ^2^Department of Ophthalmology, Medical University of Białystok, M. Sklodowska-Curie 24A STR, 15-276 Białystok, Poland

## Abstract

**Background:**

To compare the effectiveness and safety profile of ExPress implantation versus partial deep sclerectomy combined with ExPress implantation and simultaneous phacoemulsification.

**Patients and Methods:**

A prospective, randomized control study with 24-month follow-up was performed. 114 eyes were included, of which 42 eyes underwent phacoemulsification with simultaneous implantation of the Ex-Press device (ExPress), and deep sclerectomy with phacoemulsification and simultaneous implantation of the Ex-Press device (DS-ExPress) was performed in 72 eyes. The main outcome measures were intraocular pressure (IOP), corrected distance visual acuity (CDVA), the number of antiglaucoma medications, and the rate of complications. Surgical success was defined as complete (without antiglaucoma medications) with IOP ≤18 mmHg in criterion A, IOP ≤16 mmHg in criterion B, and IOP ≤12 mmHg in criterion C. Satisfactory success was defined as the same IOP levels for individual criteria with a maximum of 2 antiglaucoma medications.

**Results:**

Before the procedure, mean IOP in the ExPress group was 17.5 ± 4.7 mmHg; after 24 months, it decreased by 13% to 16.0 ± 3.0 mmHg (*P* < 0.05). In the DS-ExPress group, mean IOP reduced from 16.3 ± 4.4 mmHg to 14.3 ± 3.3 mmHg (*P* < 0.05), which was a 9% reduction compared to the initial value. In the DS-ExPress group, 65.9% of the patients did not use topical pharmacotherapy, and the same is true for 29.2% of the ExPress group (*P*=0.004).

**Conclusions:**

This modification is efficient in surgical treatment of glaucoma, especially when very low postoperative IOP is needed. A less amount of antiglaucoma medicines are needed.

## 1. Introduction

The hypotensive action of penetrating surgery with implantation of the Ex-Press device is well known and documented in the literature [1–4]. Its efficacy in reducing intraocular pressure (IOP) equals that of trabeculectomy, but with a lower percentage of intra- and postoperative complications and a higher surgical success rate [5–7]. In contrast to trabeculectomy, a block of the sclera is not excised during the procedure, and the aqueous is drained though the constant cross section of the implant; thus, it is easier to predict the degree of filtration [8]. The hypotensive effect is caused by subconjunctival drainage of the aqueous. ExPress success rates may decrease over time but are compared favourably with trabeculectomy success rates as reported in literature data [9].

Mermoud et al. [10, 11] proposed a modification of the classical surgical technique, combining deep sclerectomy (DS) with Ex-Press device implantation. An important aspect of nonpenetrating deep sclerectomy is the formation of an intrascleral decompression space during the procedure, which serves as a reservoir for the aqueous and thus enables extended aqueous absorption [10]. It is the counterpart of the subconjunctival filtering bleb, which is of lesser note in deep sclerectomy than in trabeculectomy [11]. The role of intrascleral drainage has not fully been understood, but—as Mastropasqua assumes—it performs a significant role in the hypotensive mechanisms of nonpenetrating procedures [12].

The decision to undertake surgical treatment, which is made individually for every patient, must be made at the right time during antiglaucoma therapy. Mermoud's idea provides the possibility of avoiding complications related to dissection of the filtering bleb and gives hope for achievement of better results in comparison to the classical procedure. Thanks to this, the surgical procedure could be performed earlier, even in the case of glaucoma with low IOP, where vascular factors are largely responsible for the progression of neuropathy [13, 14]. To demonstrate the potential of both types of procedures with the application of the ExPress implant, the authors decided to conduct a prospective, randomized study with a 2-year observation period concerning the efficacy, safety, and stability of effects achieved.

## 2. Patients and Methods

The project is compliant with the principles of the International Helsinki Federation for Human Rights and is aligned with the Good Clinical Practice for Trials on Medicinal Products developed by the European Union. It has been approved by the Bioethics Commission of the Military Institute of Medicine in Warsaw.

The indication for the procedure was coexisting glaucoma and cataract (NC1 and NC2) classified by means of the LOCS III scale. Patients with primary open-angle glaucoma (POAG) and normal tension glaucoma (NTG), in which a satisfactory IOP level was not achieved despite maximum tolerable hypotensive treatment, both topical and systemic, were qualified for the procedure. Additional inclusion criteria were as follows: documented progression of loss of field of vision, significant daily IOP fluctuations, no cooperation from a patient with regard to application of antiglaucoma treatment, and allergy to topical medications. Written consent to involvement and participation in the study for a period of at least 24 months was obtained from all patients after they had first been informed of the nature of the procedure and other surgical alternatives. Exclusion criteria were as follows: no consent to participation in the study, prior surgical and laser procedures in the area of the eye, narrow- or closed-angle glaucoma, postinflammatory or posttraumatic secondary glaucoma, chronic illness of the cornea or optic nerve, advanced macular degeneration, active inflammatory process, pregnancy, and systemic steroid therapy.

This prospective, randomized study included 114 eyes, of which 42 eyes underwent phacoemulsification with simultaneous implantation of the Ex-Press (ExPress) mini seton, and deep sclerectomy with phacoemulsification and simultaneous implantation of the Ex-Press (DS-ExPress) seton was performed in 72 eyes. Randomization into groups was performed by a coin flip.

## 3. Preoperative Examination

Detailed data concerning prior treatment and surgical procedures were collected from patients during qualification. Basic examinations of all patients were conducted before the procedure, covering the measurement of intraocular pressure (IOP), uncorrected distance visual acuity (UDVA), corrected distance visual acuity (CDVA), examination of the anterior and posterior eye segments, gonioscopy, and field of vision test (Humphrey 30-2 SITA Standard). IOP was measured during a preoperative examination in compliance with AGIS rules. Measurement was performed by means of a Goldmann tonometer mounted on a slit lamp. The registered results in mmHg were rounded to the nearest whole number. Every measurement was repeated twice, and if the difference between results was ≥3 mmHg, a third measurement was performed. The mean of these two or three measurements was used to determine IOP as well as for statistical analysis. IOP measurements were performed at a fixed time of day: between 8 a.m. and 10 a.m.

## 4. Surgical Technique

All procedures were conducted under retrobulbar anesthesia (2% Xylocaine and 0.5% bupivacaine) by one experienced surgeon (Rękas). In both procedures, the conjuctiva was dissected with the base at the limbus, and the sclera was exposed. A square scleral flap (5 × 5 mm) was dissected with the base at the limbus according to the technique described previously by Mermoud et al. [10]. Next, phacoemulsification was performed through a corneal incision of 2.75 mm from the temporal side using the phaco-chop technique with the help of an Infiniti Vision System camera (Alcon Surgical, Fort Worth, TX), and the IOL was implanted in the capsular bag. In the ExPress group, a mini seton was implanted at 12 o'clock according to the previously described technique [8]. In the DS-ExPress group, a deep scleral flap with dimensions of 4 × 3 mm was dissected just below the trabeculo-Descemet membrane (TDM). Next, the seton was implanted in the anterior chamber at the height of Schlemm's canal (SC), and the superficial flap was sutured above the implant. Single 10-0 nylon sutures were applied to the scleral flap (4 simple interrupted sutures), and absorbable sutures were applied to the conjunctiva.

## 5. Postoperative Protocol

During visits over the course of the observation period, IOP was measured by a Goldmann tonometer and CDVA by Snellen charts. All IOP measurements were performed between 8 a.m. and 10 a.m. The anterior chamber and eye fundus were assessed. The progression of the postoperative period was assessed accounting for complications and the number of antiglaucoma medications. Additional procedures were performed when insufficient filtration, manifested as elevated IOP (≥16 mmHg), was observed due to the absence of drainage through the seton, and also when a poorly developed or completely flat filtering bleb was observed. Insufficient filtration was diagnosed during the first month following the procedure, when healing processes still did not limit subconjunctival drainage and progressing IOP increase above 16 mmHg was observed. The presence and proper functioning of the filtering bleb and development of subconjunctival fibrosis (presence of overfilled and varicose blood vessels above the scleral flap) were assessed in detail. When fibrosis and IOP increase were determined on the basis of the aforementioned symptoms and the filtering bleb was flattened, needling was performed, as well as in cases of cystic filtering blebs. In the case of fibrosis, subconjunctival 5-FU injections were administered at a dose of 5 mg/0.2 ml. Injections were performed for the 5 following days or until fibrosis ceased and IOP stabilized, under the condition of the absence of undesired effects of antimetabolites. Suturolysis was performed within the first 2 weeks after surgery, when weak filtration through the bleb was observed because of excessively tight suturing of the scleral flap. IOP ≤6 mmHg was considered to be hypotony.

The success rate covered two categories of success: complete and satisfactory. Complete surgical success was defined as IOP ≤18 mmHg without antiglaucoma medications in criterion A, IOP ≤16 mmHg without antiglaucoma medications in criterion B, and IOP ≤12 mmHg without antiglaucoma medications in criterion C. Satisfactory success was defined as the same IOP levels for individual criteria with a maximum of 2 antiglaucoma medications. Surgical failure was defined as IOP >18 with or without antiglaucoma medications, or when the eye required further glaucoma intervention. All antiglaucoma medications were discontinued at the start of the procedure. When the procedure did not bring about the expected result, medications were resumed in accordance with EGS guidelines.

Control examinations were conducted before and after the procedure: on the 1st and 7th days and in the 1st, 3rd, 6th, 9th, 12th, 18th, and 24th months after surgery. The field of vision test was performed before the procedure and in the 6th and 12th months after surgery. All patients received an antibiotic and steroid topically for 6 weeks after the procedure. The steroid was tapered over time in the following scheme: for the first week 6 times daily, for the next two weeks 4 times daily, for the next one week 3 times daily, for the next one week 2 times daily, and for the last week 1 time a day.

Statistical analysis of the results obtained was conducted. The Shapiro–Wilk test was used to assess characteristics consistent with normal distribution, and Student's *t*-test was applied for comparisons between groups, whereas Mann–Whitney's *U* test was applied for characteristics inconsistent with this distribution. Student's *t*-test for pairs and Wilcoxon's test for pairs, accordingly, were applied when analyzing measurements at time intervals in groups. The chi^2^ independence test was applied to compare qualitative characteristics between groups. The Kaplan–Meier method with the log-rank test was applied to analyze the efficacy in the two analyzed groups.

A significance level of *P* < 0.05 was adopted in calculations as statistically significant. Calculations were carried out using the Statistica statistical package.

## 6. Results

Forty-two patients underwent the ExPress procedure, and 72 underwent Mermoud's DS-ExPress procedure. The mean observation period was 24 ± 3 months in the ExPress group and 24 ± 7 months in the DS-ExPress group. Demographic data are summarized in Table 1.

### 6.1. Intraocular Pressure

**Figure 1:**
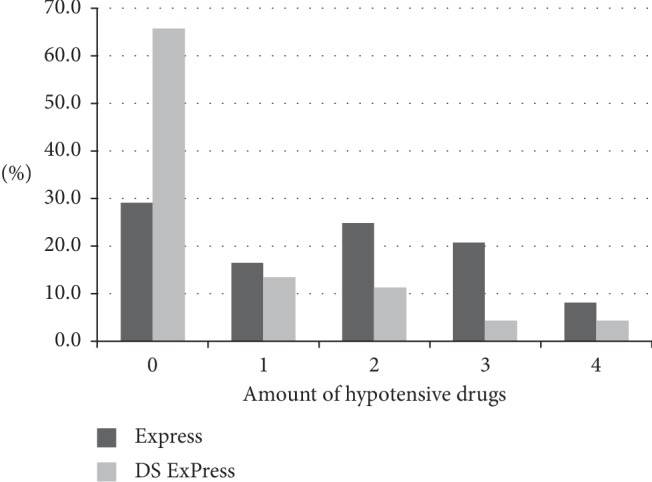
Amount of hypotensive drugs in ExPress and DS-ExPress groups at the end of the observation period (24 months).

Before the procedure, mean IOP in the ExPress group amounted to 17.5 ± 4.7 mmHg; after 24 months of observation, it decreased by 13% to 16.0 ± 3.0 mmHg (*P* < 0.05). In the DS-ExPress group, mean IOP reduced from 16.3 ± 4.4 mmHg to 14.3 ± 3.3 mmHg (*P* < 0.05), which constituted a 9% reduction compared to the initial value. In the 1st, 3rd, 9th, and 24th months after the procedure, IOP was statistically lower in the DS-ExPress group (*P*=0.029, *P*=0.049, *P*=0.001, and *P*=0.023) (Figure 1; Tables 2 and 3).

### 6.2. Antiglaucoma Medications

Before surgery, the mean number of antiglaucoma medications in the ExPress group was 2.3 ± 1.1, and after 24 months of observation, this number fell to 1.6 ± 1.3 (*P* < 0.05). In the DS-ExPress group, the mean number of medications decreased from 2.3 ± 1.1 before the procedure to a value of 0.7 ± 1.1 one year after surgery (*P* < 0.05). No statistically significant differences were observed between groups before the procedure, and after the procedure, the number of medications was lower in the DS-ExPress group (*P*=0.002) (Tables 4 and 5). Before the procedure, 82% of the ExPress group were using two or more antiglaucoma medications, as were 85% of the DS-ExPress group. A clearly marked difference between the two groups in the number of patients who did not take any medications at all became apparent at the end of the observation period (after 24 months). In the DS-ExPress group, 65.9% of the patients did not use topical pharmacotherapy, and the same is true for 29.2% of the ExPress group (*P*=0.004).

### 6.3. Surgical Success

**Figure 2:**
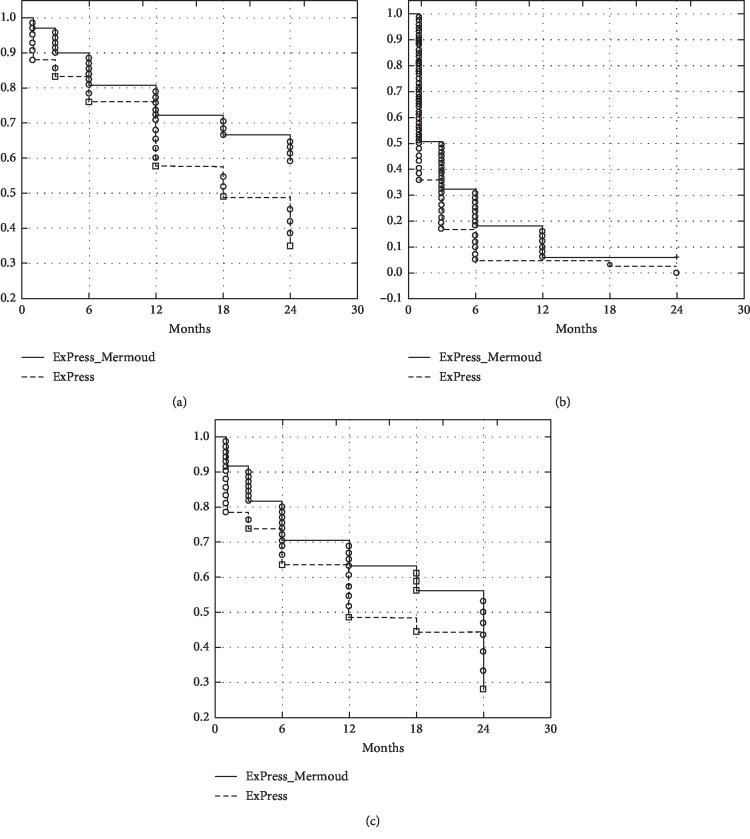
Cumulative survival proportion (%) (Kaplan–Meier) for the success criterion of intraocular pressure less than or equal to 18 mmHg (criterion A) (a), 16 mmHg (criterion B) (b), and 12 mmHg (criterion C) (c).

Complete success in criterion A (IOP ≤18 mmHg) was achieved in 42.9% of the patients in the ExPress group and 81.7% of the patients (*P*=0.019) in the DS-ExPress group; it was 42.9% and 56.3% (*P*=0.117), respectively, in criterion B (≤16 mmHg) and 0 and 13% (*P*=0.004), respectively, in criterion C (≤12 mmHg). Satisfactory success (maximum of 2 medications) in criterion A, for the ExPress and DS-ExPress groups, respectively, was 71.4% vs. 77.9% (*P*=0.151), in criterion B was 45.2% vs. 66.2 (*P*=0.004), and in criterion C was 0 vs. 15.5% (*P*=0.012) (Figure 2).

### 6.4. Corrected Distance Visual Acuity

In the ExPress group, CDVA before the procedure was 0.4 ± 0.3, and at the end of the observation period, it improved to 0.7 ± 0.3 (*P* < 0.05). In the DS-ExPress group, it amounted to 0.49 ± 0.28, increasing to 0.8 ± 0.2 (*P* < 0.05) after the procedure. No statistically significant differences between groups were observed after the procedure (*P* > 0.05) (Table 6).

In the final phase of observation in the ExPress group, the loss of 1 line on the Snellen chart was observed in 4 patients (8.7%). In 15.2% of the patients, CDVA was maintained at the level as that before the procedure. CDVA improved from 1 to 9 lines on the Snellen chart in 76.1% of the patients. Deterioration of vision in this group was caused by secondary cataract in 1 case and macular edema induced by chronic hypotony in 1 case.

In the DS-ExPress group, CDVA reduction by 1 Snellen line was observed in 7 patients (17.9%), and visual acuity was unchanged in 7.6% of the patients and increased from 1 to 9 Snellen lines in 74.3% of the patients. Secondary cataract, epiretinal membrane, and dry AMD were among the causes of deteriorated visual acuity.

### 6.5. Complications and Additional Procedures

Subconjunctival 5-FU injections were administered in 29 cases (69%) in the ExPress group and in 54 cases (75%) in the DS-ExPress group (*P*=0.412). The mean dose of 5-FU administered was 21.8 ± 7.3 mg (range 5–25 mg) in 4.3 injections on average in the ExPress group and 19.3.1 ± 8.3 mg (range 5–35 mg) in 3.8 injections on average in the DS-ExPress group (*P*=0.41). Needling was performed in 22 patients (23.8%) in the ExPress group and 16 patients (11%) in the DS-ExPress group (*P*=0.08). Laser suturolysis was performed in 21 patients (50%) in the ExPress group and 63 patients (61%) in the DS-ExPress group (*P*=0.274). An additional sealing suture was applied in one patient in the ExPress group (*P*=0.274). Two patients from the ExPress group underwent reoperation: one because of extrusion of the mini seton by the scleral flap and the other because of occlusion of the filtering bleb. Classical trabeculectomy was performed in both cases. The patients were disqualified from the program (their results prior to this were not disqualified from the database). One patient from the DS-ExPress group underwent reoperation because of the absence of IOP regulation. Symptoms of malignant glaucoma were observed in one female patient in the ExPress group, which occurred in the 6th and 8th months after surgery. After the application of cycloplegics and a pressure dressing, the symptoms ceased without any additional surgical procedures (Table 7).

## 7. Discussion

Deep sclerectomy represents a group of nonpenetrating procedures that were developed in order to improve the safety of fistular antiglaucoma operations [15, 16]. This goal was achieved, thanks to avoidance of a full-wall connection with the anterior chamber and the creation of a decompression space that serves as a reservoir for the aqueous, making its extended absorption possible [17]. This affords effective IOP control after the procedure and prevents the consequences of long-term hypotony [18, 19]. Based on ultrasound biomicroscopy tests, 3 potential aqueous drainage sites that could be correlated with IOP reduction following DS were found: the intrascleral decompression space, subconjunctival space, and suprachoroidal space [20, 21]. Numerous reports indicate that deep sclerectomy has a higher degree of safety compared to trabeculectomy, but also a lower hypotensive efficacy [15, 17].

The ExPress implant, created as an alternative to trabeculectomy, has hypotensive action similar to this procedure, but also a better safety profile. After ExPress implantation, the conjunctival incision is smaller since only a zone of 2 hours on the clock is required to position the seton properly, and because of this, it is possible to place the implant in eyes with coexisting conjunctival scars [1]. Another benefit of the ExPress implantation procedure is that it avoids the need for sclerectomy that arises during trabeculectomy since the self-sealing opening made by a 27 G needle maintains the stability of the anterior chamber without the necessity of applying viscoelastics [22]. The small interior diameter of the ExPress implant significantly limits the occurrence of hypotony induced by excessive filtration, which reduces the number of cases of anterior chamber shallowing [23]. Another feature speaking of the advantages of ExPress is the fact that iridotomy is not performed over the course of its implantation, thus reducing the risk of bleeding, inflammation, and pigment release [6]. Because of this, the number of cases of filtering bleb revision is reduced, as is the intensity of steroid drop administration after the procedure [24]. In both of these procedures, the creation of subconjunctival drainage is the IOP-reducing mechanism [25].

In 2011, Mermoud et al. [26] proposed a modification of the classical method of Ex-Press seton implantation, based on the additional creation of an intrascleral lake through excision of the deep flap below the trabeculo-Descemet membrane (TDM). The aqueous flows directly from the anterior chamber into the intrascleral space through the hole in the implant, from where it is drained into the subconjunctival space and collector channels. This method potentially creates four paths for aqueous drainage. They are the subconjunctival filtering bleb, intrascleral filtering bleb, suprachoroidal drainage, and intrascleral drainage through the inlets of Schlemm's canal. This may result in great IOP reduction relative to that after classical ExPress implantation, which creates only one drainage path [17]. Mermoud in his studies observed a 27% IOP reduction relative to initial values and surgical success at a level of 45.6% (complete) and 85.2% (satisfactory).

The results we have obtained seem to confirm the validity of Mermoud's assumption. Even at an early postoperative period, the IOP value in the DS-ExPress group was lower than that in the ExPress group, and it reached a level of statistical significance after 1, 3, and 9 months (*P*=0.029, *P*=0.049, and *P*=0.001). Even after 24 months, pressure in the DS-ExPress group and the number of medications were statistically lower than those in the ExPress group. Complete and satisfactory surgical success in criterion C (up to 12 mmHg) was also statistically greater in the DS-ExPress group than in the ExPress group (12.7% vs. 0%, *P*=0.012, and 15.5% vs. 0%, *P*=0.004). Moreover, the number of patients with IOP ≤16 mmHg and a maximum of two medications (criterion B) was significantly higher in the DS-ExPress group (66.2% vs. 45.2%, *P*=0.024). This means a greater number of patients achieved very low pressure, which is particularly desirable in cases of advanced glaucoma neuropathy. This may indicate an “overlap” in the action of various hypotensive mechanisms in the DS-ExPress group. The risk of conjunctival fibrosis increases proportionally to the time that passes from the procedure, which may translate to reduction of the hypotensive effect in the ExPress group. In the DS-ExPress group, despite conjunctival fibrosis, the hypotensive component of the intrascleral lake is still maintained, which is probably responsible for maintaining the desired IOP. The presence of an intrascleral lake also increases the percentage of patients with better IOP control.

There are not many reports in the literature on the subject of long-term results after implantation of the Ex-Press seton in a large group of patients. There are also few observations with an observation period lasting more than 24 months [27, 28]. Mariotti et al. [9] presented the results of a retrospective study on a group of 248 patients with an observation period up to 7 years (3.4 ± 1.8). Surgical success, defined as 5 < IOP < 18 mmHg, dropped by 5% annually on average after the procedure, from 83% and 85% after 1 year to 57% and 63% after 5 years. De Jong reports a reduction of success from 86.8% after the first year to 59% after five years. In the tube vs. trabeculectomy (TVT) study, surgical success following trabeculectomy after the first year amounted to 83% and after 5 years to 46.4%.

The data on long-term observation of DS are surprising. Bissig et al. demonstrated surgical success at 91% after 8 years and 89% after 10 years [11]. Similarly, in a study with a 5-year observation period, Mercecia et al. noted satisfactory success at a level of 89.1% after 2 years and 80% after 5 years and complete success at 81.2% and 68.3%, respectively, for a success criterion of IOP <19 mmHg [16]. The roles of individual hypotensive mechanisms in nonpenetrating procedures are not fully known and are broadly discussed in the literature. Perhaps, it is the presence of the filtration lake that is responsible for more permanent effectiveness of the procedure.

It is difficult to compare the surgical success we have achieved to that of Mermoud's study since he applied an observation period that was twice as long as ours. After 48 months, 38.9% of his patients without medications and 66% with medication had IOP <15 mmHg. He observed IOP <18 mmHg in 45.6% of the patients without medications and in 85.2% with medications. He noted a 25.4% drop in IOP after 24 months and a 27% drop after 48 months. In our group, after 24 months of observation, 56.3% of the patients without medications and 66.2% with medications had IOP ≤16 mmHg, and IOP ≤18 mmHg was achieved by 64.8% patients without medications and 77.9% with medications. The percentage of IOP reduction in comparison with the initial value amounted to 13%; however, it is difficult to estimate the precise value of IOP reduction since the study was conducted without a prior wash-out period, and 18 patients in both groups were taking 4 or more antiglaucoma medications because of advanced glaucoma neuropathy (including oral carbonic anhydrase inhibitors) within a short period prior to the procedure. In addition, the IOP level before the procedure was lower in our groups (17.6 ± 4.7 mmHg in ExPress and 16.3 ± 4.4 in DS-ExPress vs. 18.1 ± 5.3 mmHg in Mermoud's work) since we accounted for NTG patients. The type of implant was also different—a P-50 implant was used in our study, while an X-50 implant was used in Mermoud's. As shown by later studies, this has no impact on the flow resistance of aqueous through the interior opening [29]. The mean number of antiglaucoma medications in both studies after 24 months is also comparable (0.6 ± 0.9 vs. 0.7 ± 1.1). In the authors' study, 65.9% of the patients in the DS-ExPress group did not take any antiglacuoma medications (29.2% in the ExPress group; *P*=0.004), and in relation to this, it can be presumed that the creation of an intrascleral lake improves the effectiveness of the procedure and improves IOP control.

Our results with the application of the ExPress implant by itself were similar to the results from previous studies. For the Ex-Press mini seton implantation operation, Dahan and Carmichael [2] reported pressure at 14.5 ± 5.0 mmHg after 12 months and 14.2 ± 4.2 mmHg after 24 months, Coupin et al. [30] reported pressure at 14.0 ± 2.0 mmHg after 6 months and 14.3 ± 2.3 mmHg after 12 months, and Konopinska et al. [4] reported pressure at 14.9 ± 3.6 mmHg after 6 months and 17.1 ± 5.0 mmHg after 12 months. In this article, IOP in the ExPress group was 14.3 ± 3 mmHg after 6 months, 15.3 ± 3.5 mmHg after a year, and 16±3 mmHg after 24 months.

The TDM, with a thickness of 0.25 ± 0.09 mm, through which aqueous is filtered, is undisturbed during DS after the removal of the roof above Schlemm's canal. According to Karlen et al., in as many as 30% of cases, the TDM is perforated during dissection of the anterior part of the deep flap in the clear cornea, particularly over the course of a learning curve [31]. Mermoud's modification minimizes surgical risk but does not force resignation from the advantages of the mechanism of aqueous resorption from the intrascleral space.

This technique's greatest flaw is that it is time-consuming and requires experience from the surgeon. The Ex-Press mini seton was assumed to simplify antiglaucoma procedures, but in combination with the proposed modification, it is a decidedly more complicated procedure. Furthermore, deep sclerectomy was done in an attempt to avoid the risk of postoperative hypotony, which may reach 4.5–25% in penetrating procedures [32]. The introduction of the ExPress implant's tip into the anterior chamber may cause postoperative hypotony, the risk of which is minimized in nonpenetrating procedures. In our case, this occurred in 14 patients (23.4%), whereas this occurred in 4 patients (8%) in Mermoud's trial. In relation to this, it seems beneficial sometimes to employ the modification of the ExPress implantation procedure proposed by Mermoud, which has a similar safety profile, and as it turns out, it also brings about beneficial effects when it is necessary to reach a very low IOP after surgery.

In order to minimize side effects and improve the IOP-reducing potential of antiglaucoma procedures, more and more surgical methods are sought. It seems that the modification proposed by the authors makes it possible to combine the advantages of Ex-Press seton implantation and eliminate the potential risks of TDM perforation associated with deep sclerectomy. It is safer and more effective than classical procedures, and however, it demands high skill from the surgeon. To decide which procedure is more beneficial, studies on a larger group of patients and with a longer observation period are required. Our results serve as a supplement to the discussion about the role of the intrascleral lake.

## Figures and Tables

**Table 1:** Patients' demographic data.

Group		ExPress	DS-ExPress	^*∗*^ *P*
Follow-up (months)		24 ± 3	24 ± 7	0.91
*n*		42	72	—
Age (years)		71 ± 9	73 ± 5	0.82
Sex (female/male)		25/17	38/34	—
Eye (right/left)		20/22	35/37	—
Glaucoma type	POAG	29	54	<0.05
	NTG	13	18	

ExPress: Ex-Press device implantation group; DS-ExPress: Mermoud's ExPress modification group; POAG: primary open-angle glaucoma; NTG: normal tension glaucoma. ^*∗*^Student's *t*-test or *χ*^2^ test.

**Table 2:** Intraocular pressure (IOP): mean values, median values, standard deviations, and range in the ExPress and DS-ExPress groups at specific times after surgery.

Time	ExPress			DS-ExPress			^*∗*^ *P*
	Mean ± SD	Median	Range	Mean ± SD	Median	Range	
Pre-op	17.6 ± 4.7	16.50	10–28	16.3 ± 4.4	16.00	8–30	0.152
1st day	7.9 ± 3.8	6.50	4–18	8.6 ± 3.4	9.00	4–17	0.154
7th day	8.6 ± 3.2	9.50	4–16	9.3 ± 5.1	8.50	4–38	0.910
1st month	14.5 ± 4.4	13.50	10–30	13.1 ± 3.8	13.00	8–30	0.029
3rd month	14.0 ± 2.9	14.00	10–23	13.1 ± 3.7	12.00	6–30	0.049
6th month	14.6 ± 3.1	14.00	10–26	13.7 ± 3.0	13.00	10–21	0.061
9th month	15.2 ± 2.9	14.50	11–26	13.5 ± 3.2	13.00	9–28	0.001
12th month	15.4 ± 3.6	15.00	10–25	14.1 ± 2.4	14.00	10–20	0.122
18th month	14.8 ± 2.5	14.00	10–21	14.0 ± 2.6	14.00	8–20	0.59
24th month	16.0 ± 3.0	16.00	10–22	14.3 ± 3.3	13.50	9–23	0.023

ExPress: Ex-Press device implantation group; DS-ExPress: Mermoud's ExPress modification group; SD: standard deviation; pre-op: before operation. ^*∗*^Mann–Whitney's *U* test.

**Table 3:** Intraocular pressure (IOP) in the ExPress and DS-ExPress groups at the end of the observation period.

IOP over time	ExPress group	DS-ExPress group	*P*
*N*	42	72	
IOP ≤18 mmHg, *n* (%)	18 (42.9)	46 (64.8)	0.019
	30 (71.4)^*∗*^	58 (81.7)^*∗*^	0.151
IOP ≤ 16 mmHg, *n* (%)	18 (42.9)	24 (56.3)	0.117
	19 (45.2)^*∗*^	45 (66.2)^*∗*^	0.024
IOP ≤ 12 mmHg, *n* (%)	0 (0)	9 (12.7)	0.012
	0 (0)^*∗*^	11 (15.5)^*∗*^	0.004

^*∗*^With a maximum of 2 antiglaucoma agents.

**Table 4:** Amount of hypotensive drugs: mean values, median values, standard deviations, and range in the Ex-Press device implantation (ExPress) and Mermoud's ExPress modification (DS-ExPress) groups at specific times after surgery.

Time	ExPress			DS-ExPress			^*∗*^ *P*
	Mean ± SD	Median	Range	Mean ± SD	Median	Range	
Pre-op	2.3 ± 1.1	2.0	1–5	2.3 ± 1.1	2.0	1–5	0.79
6th month	0.37 ± 0.8	0	0–4	0.32 ± 0.8	0	0–4	0.543
12th month	0.7 ± 1.1	0	0–4	0.51 ± 1.0	0	0–4	0.193
24th month	1.6 ± 1.3	2	0–4	0.7 ± 1.1	0	0–4	0.002

Pre-op: before operation. ^*∗*^Mann–Whitney's *U* test.

**Table 5:** Amount of hypotensive drugs in the Ex-Press device implantation (ExPress) and Mermoud's ExPress modification (DS-ExPress) groups at 2 years after surgery.

Name of the procedure		Number of medications 2 years after surgery					Total
		Pre-op	1	2	3	4	
ExPress	*N*	7	4	6	5	2	24
	%	29.2	16.7	25.0	20.8	8.3	100.0
DS-ExPress	*N*	29	6	5	2	2	44
	%	65.9	13.6	11.4	4.5	4.5	100.0
Total	*N*	36	10	11	7	4	68
	%	52.9	14.7	16.2	10.3	5.9	100.0
*P*	0.004		>0.05	>0.05	>0.05	>0.05	

Pre-op: before operation. ^*∗*^Mann–Whitney's *U* test.

**Table 6:** Visual acuity (Snellen charts): mean values, median values, standard deviations, and range in the ExPress and DS-ExPress groups at specific times after surgery.

Time	ExPress			DS-ExPress			^*∗*^ *P*
	Mean ± SD	Median	Range	Mean ± SD	Median	Range	
Pre-op	0.45 ± 0.3	0.5	0–2.8	0.49 ± 0.3	0.5	0–3	0.436
1st day	0.29 ± 0.2	0.2	0–2	0.43 ± 0.2	0.4	0.05–2	0.005
7th day	0.45 ± 0.2	0.5	0–2	0.59 ± 0.2	0.6	0–2	0.013
1st month	0.63 ± 0.3	0.6	0–1.7	0.73 ± 0.2	0.8	0–2	0.089
3rd month	0.78 ± 0.2	0.9	0–1.4	0.78 ± 0.2	0.8	0–1.7	0.665
6th month	0.86 ± 0.2	0.9	0–1.7	0.83 ± 0.2	0.9	0–1.7	0.433
9th month	0.82 ± 0.2	0.9	0–1.7	0.81 ± 0.2	0.9	0–1.7	0.74
12th month	0.81 ± 0.2	1.0	0–1.7	0.81 ± 0.3	0.9	0–1.4	0.752
18th month	0.76 ± 0.3	0.8	0–0.4	0.8 ± 0.2	0.8	0–0.4	0.872
24th month	0.77 ± 0.3	0.8	0–0.4	0.8 ± 0.2	0.9	0–0.4	0.734

ExPress: Ex-Press device implantation group; DS-ExPress: Mermoud's ExPress modification group; SD: standard deviation; Pre-op: before operation. ^*∗*^Mann–Whitney's *U* test.

**Table 7:** Postoperative complications.

Complications	ExPress, *n* (%)	DS-ExPress, *n* (%)	^*∗*^ *P*
Hyphema	—	1 (1.7)	0.331
Fibrosis	11 (19.3)	13 (16.4)	0.753
Anterior chamber cells	3 (5.3)	4 (6.7)	0.752
Hypotony	12 (21.1)	14 (23.3)	0.819
Choroidal detachment	9 (15.8)	7 (11.7)	0.521
Macular edema	2 (3.5)	1 (1.7)	0.532
Shallow anterior chamber	4 (7.0)	6 (10.0)	0.568

ExPress: Ex-Press device implantation group; DS-ExPress: Mermoud's ExPress modification group. ^*∗*^*χ*^2^ test.

## Data Availability

The data used to support the findings of this study are included within the article.
